# Comparison of reversal of rat pulmonary fibrosis of nintedanib, pirfenidone, and human umbilical mesenchymal stem cells from Wharton’s jelly

**DOI:** 10.1186/s13287-020-02012-y

**Published:** 2020-11-30

**Authors:** Kuo-An Chu, Chang-Ching Yeh, Fu-Hsien Kuo, Wen-Ren Lin, Chien-Wei Hsu, Tien-Hua Chen, Yu-Show Fu

**Affiliations:** 1 Division of Chest Medicine, Department of Internal Medicine, Kaohsiung Veterans General Hospital, Kaohsiung, Taiwan, Republic of China; 2 Institute of BioPharmaceutical Sciences, National Sun Yat-sen University, Kaohsiung, Taiwan, Republic of China; 3 Department of Obstetrics and Gynecology, Taipei Veterans General Hospital, Taipei, Taiwan, Republic of China; 4 Institute of Clinical Medicine, National Yang-Ming University, Taipei, Taiwan, Republic of China; 5 Department of Obstetrics and Gynecology, National Yang-Ming University, Taipei, Taiwan, Republic of China; 6 Institute of Anatomy and Cell Biology, School of Medicine, National Yang-Ming University, Taipei, Taiwan, Republic of China; 7 Trauma Center, Department of Surgery, Veterans General Hospital, Taipei, Taiwan, Republic of China; 8 Division of General Surgery, Department of Surgery, Veterans General Hospital, Taipei, Taiwan, Republic of China; 9 Department of Anatomy and Cell Biology, School of Medicine, National Yang-Ming University, No. 155, Sec. 2, Li-Nung Street, 112, Taipei, Taiwan, Republic of China

**Keywords:** Pulmonary fibrosis, Human umbilical mesenchymal stem cells, Nintedanib, Pirfenidone

## Abstract

**Background:**

The present study compared the effects of antifibrotic medications, pirfenidone, and nintedanib, with transplantation of human umbilical mesenchymal stem cells (HUMSCs) in restoring rat pulmonary fibrosis (PF).

**Methods:**

A stable animal model was established via an intratracheal injection of 5 mg bleomycin (BLM). One single transplantation of 2.5× 10^7^ HUMSCs or initiation of daily oral nintedanib/pirfenidone administration was performed on day 21 following BLM damage.

**Results:**

Pulmonary function examination revealed that BLM rats exhibited a significant decrease in blood oxygen saturation and an increase in respiratory rates. While no significant improvements were found in BLM rats receiving nintedanib or pirfenidone, those who transplanted with HUMSCs showed a statistical amelioration in blood oxygen saturation and significant alleviation in respiratory rates. Quantification results revealed that a significant reduction in alveolar space and marked increases in substantial cell infiltration and collagen deposition in the left lungs of BLM rats. No significant alteration was observed in BLM rats administered nintedanib or pirfenidone. However, BLM rats transplanted with HUMSCs had a significant recovery in alveolar space and noticeable decreases in cell infiltration and collagen deposition. The inflammatory cell numbers in the bronchoalveolar lavage was increased in the BLM group. While the rats treated with nintedanib or pirfenidone had a lower cell number than the BLM group, a higher cell number was found as compared with the Normal group. In rats transplanted with HUMSCs, the cell number did not differ from the Normal group.

**Conclusions:**

Transplantation of HUMSCs could effectively treat PF as opposed to the administration of anti-fibrotic drugs with nintedanib or pirfenidone with a significant better result in lung volume, pathological changes, lung function, and blood oxygen saturation.

## Introduction

A number of risk factors can result in the destruction of the lung tissues. Lung impairment triggers the number of functional alveoli to decrease, and the alveolar compartments are gradually replaced by fibrotic tissues, leading to the development of pulmonary fibrosis (PF). Damages caused by lung fibrosis are irreversible, with progressive deterioration of lung functionality. Hence, when the severity of lung fibrosis worsens, patients would suffer respiratory failure and eventually, death. Once diagnosed with lung fibrosis, the median survival of patients is generally less than 3 years [1, 2].

A variety of animal models have been established for PF in basic research [3–8]. Among all the models, intratracheal injection of BLM was most extensively applied due to its relatively short period of disease progression, and the pathological condition closely resembles patients with idiopathic PF. However, the severity of damage caused by the intratracheal injection of BLM to each lobe of the lung varies from experiment to experiment. In addition, the pathological changes of the appearance of left and right lungs are not apparent enough for observation, and the changes in blood oxygen saturation could not be detected by an oximeter. The above reasons bring difficulties in applying this model for the evaluation of the therapeutic effects of stem cell transplantation. In order to precisely evaluate the effect of medications or xenografted stem cells in treating PF in rat, our laboratory has previously established a severe, reproducible, consistent, one-sided left-lung fibrosis animal model, which provides advantages in keeping the rat alive and allowing accurate evaluations of the therapeutic effects of human umbilical mesenchymal stem cells from Wharton’s jelly (HUMSCs) for rat PF [9].

Pirfenidone has an anti-inflammatory effect by inhibiting the expressions of proinflammatory cytokines [10]. In vivo experiments revealed that pirfenidone not only resulted in reduced gene and protein expressions of PDGF but also inhibited gene overexpression of procollagen in BLM-induced PF, suggesting that pirfenidone could prevent the lung from developing fibrosis [11–15]. However, no evidence currently supports the application of pirfenidone in reversal of PF.

Nintedanib was initially used as an anti-cancer agent. It is a tyrosine kinase inhibitor that inhibits tyrosine phosphorylation on PDGF, VEGF, and FGF receptors and the activation of their downstream pathways. Therefore, nintedanib could effectively suppress inflammation, angiogenesis, and fibroblast activation [16–18]. However, no proofs presently suggest that nintedanib could be used for the reversal of PF.

Nevertheless, in clinical practice, most patients with PF attending hospitals have already developed various degrees of respiratory symptoms. Therefore, reversing the functionality and status of PF is an urgent and important issue. Our laboratory previously established a severe, reproducible, consistent, one-sided left-lung PF animal model. While intratracheal transplantation of 2.5× 10^7^ HUMSCs was performed on day 21 after BLM injection, significant therapeutic effects on lung functionality and tissue morphology were observed at 1 month post-transplantation [9].

Therefore, we applied the severe, reproducible, consistent, one-sided left-lung PF animal model in this study. Initiation of consecutive pirfenidone/nintedanib administration or single intratracheal transplantation of 2.5× 10^7^ HUMSCs was performed on day 21 post-BLM injection. Our objective was to compare their effects in reversal of PF.

## Materials and methods

### Establishment of left-lung PF animal model

Eight-week-old Sprague-Dawley (SD) rats weighed around 250 g were provided by the Laboratory Animal Center (LAC) of National Yang-Ming University. Rats are prone to death after BLM administration if rats were too young or underweight. It will take more time to feed and care if rats are too old or overweight. Moreover, the limitation of the MRI scan cabin could not afford rats with heavy body weight. Therefore, rats weighing 250 g were selected as the experimental animals. Following confirmation of anesthesia depth, male SD rats received 5 unit/5 mg BLM/250 g body weight (Nippon Kayaku Co., Ltd.) in a 200 μl phosphate-buffered saline by intratracheal injection and were then rotated to the left side by 60° for 90 min to establish a severe, reproducible, left-lung PF animal model [9].

### Administration of pirfenidone with oral gavage

The anti-fibrotic agent (brand name: PIRESPA® Tablet 200 mg, Shionogi Pharma Co., Ltd., MHW Medicine Import No.: 026734) contains 200 mg pirfenidone (5-methyl-1-phenyl-2-1H-pyridine-2-one). Dose conversion from a 50 kg adult human to a 400 g adult rat was performed, and each rat was to receive 1.6 mg of pirfenidone. A tablet of pirfenidone was dissolved in 50 ml of reagent grade water, and each rat was then administered with 1.6 mg/0.4 ml pirfenidone.

On day 21 after BLM injection, 1.6 mg/0.4 ml pirfenidone was administered twice daily for 2 weeks. From the third week and onwards, 3.2 mg/0.8 ml was given twice daily for another 2 weeks until day 49 after BLM injection.

### Administration of nintedanib with oral gavage

The Ofev^Ⓡ^ capsule (Catalent Germany Eberbach GmbH, MHW Medicine Import No.: 026568) contains 150 mg of nintedanib. Dose conversion from a 50 kg adult human to a 400 g adult rat was conducted, and each rat was to receive 1.2 mg of nintedanib. A capsule of nintedanib was dissolved in 50 ml of reagent grade water, and each rat was then administered with 1.2 mg/0.4 ml of nintedanib.

On day 21 after BLM injection, 1.2 mg/0.4 ml of nintedanib was administered twice daily for 4 weeks until day 49 after BLM injection.

### Isolation, culture, and transplantation of HUMSCs

The use of the human umbilical cords in this study was approved by the Research Ethics Committee of Taipei Veterans General Hospital (2018-01-020 AC). The human umbilical cords were collected and kept at 4 °C Hank’s Balanced Salt Solution (HBSS). In a laminar hood, the umbilical cords were disinfected by soaking in 75% ethanol and were then placed in the HBSS solution. Subsequently, the mesenchymal tissue (Wharton’s jelly) was cut into small pieces and centrifuged at 4000 rpm for 5 min. After removal of the supernatant fraction, the umbilical mesenchymal tissue was treated with collagenase and trypsin, followed by the addition of a fetal bovine serum (FBS; Gibco 10437-028) to stop the reaction; at that point, the umbilical mesenchymal cells were fully processed into HUMSCs. Finally, HUMSCs were then used directly for cultures in 10% FBS Dulbecco’s modified Eagle’s medium (DMEM) or stored in liquid nitrogen for later use. HUMSCs were collected between the tenth and fifteenth passages for transplantation into rats in this study. HUMSCs were found to express high levels of matrix receptors (CD44 and CD105) but did not express HLA-DR. Meanwhile, osteogenic and neuronal differentiation of HUMSCs at the tenth generation all succeeded (Supplemental Figure 1A and B).

HUMSCs were treated with 0.05% Trypsin-EDTA (Gibco 15400-054) for 2.5 min. Cells were then collected, washed twice with 10% FBS DMEM, and centrifuged at 1500 rpm for 5 min, and the supernatant was removed. The pelleted cells were then suspended in a concentration of 2.5 × 10^7^ in 200 μl of sterile saline solution. On day 21 after intratracheal BLM, rats were treated with 2.5 × 10^7^ HUMSCs by intratracheal transplantation.

### Animal groups

**Fig. 1:**
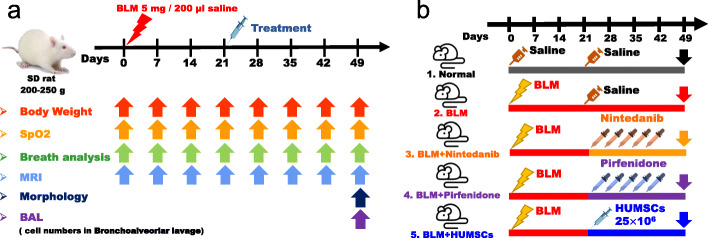
Experimental flowchart for inducing PF in rats’ left lung, administration of therapeutic agents nintedanib and pirfenidone, and transplantation of HUMSCs. The day when 5 mg BLM was injected into rats’ left bronchus was set as day 0. On day 21 following BLM injection, either daily administration of nintedanib/pirfenidone was initiated or one single transplantation of HUMSCs was conducted. After 4 weeks, the changes in the lung were observed, and various assessments were performed (**a**). The rats were divided into a total of five groups (**b**)

The animals were randomized to the following five treatment groups:

1. Normal group: Rats received a saline injection. On day 21 after injection, only 200 μl of saline was intratracheally administered (Fig. 1a).
2. BLM group: Rats received 5 mg BLM injection. On day 21 after BLM injection, no treatment but saline was intratracheally administered (Fig. 1a).
3. BLM + Nintedanib group: Rats received BLM injection. On day 21 after BLM injection, nintedanib was orally administered twice per day for 4 weeks. The rats were sacrificed on day 49 (Fig. 1a).
4. BLM + Pirfenidone group: Rats received BLM injection. On day 21 after BLM injection, pirfenidone was orally administered twice per day for 4 weeks. The rats were sacrificed on day 49 (Fig. 1a).
5. BLM + HUMSCs group: Rats received BLM injection. Intratracheal transplantation of 2.5 × 10^7^ HUMSCs was performed on day 21 after BLM injection. The rats were sacrificed on day 49 (Fig. 1a).

Animals were randomized, blinded for treatments and assays. Weight, blood oxygen saturation, respiratory rate, and MRI were assessed weekly. During the measurement, rats were put on a heating pad (36 ± 2 °C) and the room temperature is maintained at a constant temperature (22 ± 2 °C). Rats were sacrificed on day 49 after BLM administration for observing the morphology of the lung and for bronchoalveolar lavage cell counts (Fig. 1b).

### Pulmonary function testing

#### Arterial blood oxygen saturation

After shaving off the fur on the rats’ front legs, they were anesthetized with isoflurane (Baxter 228–194). The shaved legs were then clipped with a pulse oximeter (Pulseoximeter, Rossmax SB100) for measuring arterial blood oxygen saturation.

#### Pulmonary respiratory rates

The rats were placed in a closed cylinder-shape detection chamber (emka Technologies, whole body plethysmograph), and the changes in breath flow during 15 min were recorded by the software BIOPAC BSL 4.0 MP45. The resting respiratory rate of rats was then quantified.

### Magnetic resonance imaging (MRI)

Rat lung images were obtained using the MRI (BRUKER BIOSPEC 70/30) at the Instrumentation Center, National Taiwan University. The thoracic cavity of rats was scanned and photographed horizontally from rostral to caudal every 1.5 mm until the whole cavity was completely scanned. Because the first image obtained in each rat varied in position, the total number of images in the horizontal plane was between 20 and 25.

In order to reduce the bias due to the different number of total images when quantifying the volume of the left lung, the carina of the trachea was used as a landmark for image positioning. In addition to the slice containing the carina, two slices before and after the carina were collected. These five images were summed for the quantification of the black alveolar space to represent the left-lung alveolar volume of each rat (Supplemental Figure 3–7).

### Sacrifice and perfusion fixation of experimental animals

Animals were anesthetized and then perfused with 0.01 M PBS. Both lungs were removed and immersed in a fixation solution with 4% paraformaldehyde (Sigma 10060) and 7.5% picric acid (Sigma 925-40). The lungs were post-fixed in the fixative solution and then subjected to paraffin embedding. Lung tissue blocks were sectioned into 5 μm slices. A serial sagittal section was performed from the outermost lateral side. Ten slices were numbered consecutively and placed on slides for various immunohistochemistry (IHC) examinations (Supplemental Figure 2).

### Hematoxylin and eosin (H&E) staining

Lung tissue sections were immersed in hematoxylin solution (Muto Pure Chemicals Co., Ltd.; No. 3008-1) and eosin solution (Muto Pure Chemicals Co., Ltd.; No. 3200-2). The left lung volume, percentage of cell infiltration area, and air space (Supplemental Figure 2, Column A), stained using H&E, were quantified by the average red signals in every left lung section (infiltration and air space). The total left-lobe volume was the summation of all H&E signals in the collected images. The quantification method for alveoli circumference was based on the inner space peripheral lengths of each empty alveoli in the unit area (*N* = 8 or 9, 30 images each), analyzed with Image-Pro software.

### Sirius red stain

Lung tissue sections were stained in 0.1% Sirius red (Sigma 2610-10-8) in picric acid and then photographed using optical microscopy. The percentage of collagen deposition (Supplemental Figure 2, Column B), stained by Sirius red, was quantified by the average number of red signals in every left lung section. Image signals (*N* = 8 or 9, 30 images each) were analyzed with Image-Pro software.

### Immunohistostaining

Lung slices (with recovered antigens) were reacted with primary antibodies (mouse anti-α-smooth muscle actin antibody for myofibroblast [SMA, Sigma A2547]; mouse anti-ED1 antibody for total macrophage [Millipore MAB1435]; rabbit anti-CD86 antibody for M1 macrophage [Proteintech, 13395-1-AP]; mouse anti-CD163 antibody for M2 macrophage [BioRad MCA342R]; rabbit anti-NADPH oxidase antibody [Abcam, ab133303]; mouse anti-catalase antibody [Abnova, H00000847-A01]) at 4 °C for 12–18 h and then reacted with secondary antibodies. Samples were then reacted with avidin-biotinylated-horseradish peroxidase complex (ABC Kit, Vector Laboratories) and finally developed with DAB. Finally, the numbers of M1 macrophage or M2 macrophage were counted from ten optic fields in three left lung sections of each group.

### Quantitative real-time PCR

Extraction of total RNA from the lung was performed with RNAiso Plus reagent and further reverse-transcribed using a PrimeScript RT reagent kit (BIONOVAS HiScript I First Strand cDNA Synthesis Kit, AM0675-0050). SYBR-Green mix (Luna Universal qPCR Master Mix, M3003) was used to carry out quantitative PCR according to the manufacturer’s instructions. Target gene expression was normalized to β-actin levels in respective samples as an internal control and calculated using the 2−ΔΔCq method, and the relative mRNA expression was further calculated through normalizing to the Normal group.

### Rat GAPDH

F: 5′-CTCTACCCACGGCAAGTTCAAC-3′. R: 5′-GGTGAAGACGCCAGTAGACTCCA-3. Product length: 160 bps.

### Rat collagen type1 alpha 1 chain (*Col1a1*)

F: 5′-TCCTGCCGATGTCGCTATC-3′. R: 5′-CAAGTTCCGGTGTGACTCGTG-3′. Product length: 234 bps.

### Western blotting

Lung tissue samples from each group were loaded separately into individual wells. The polyvinylidene fluoride (PVDF) paper was reacted with primary antibodies (mouse anti-α-SMA antibody, rabbit anti-MMP-9 antibody [Abcam ab76003], mouse anti-Toll-like receptor-4 antibody [TLR-4, Abcam ab30667], and mouse anti-β-actin antibody [Sigma A5411] for internal control) at 4 °C for 12–18 h. Subsequently, the membrane was reacted with the corresponding secondary antibodies at room temperature for 1 h. The protein bands were quantified using ImageJ software and normalized using individual internal controls for comparison.

### Bronchoalveolar lavage

Rats were anesthetized and their airways were lavaged two times with 2 ml saline/each, and 1 ml bronchoalveolar lavage fluid (BALF) was recovered. Total cell counts were determined using a hemocytometer.

### Statistical analysis

All data are presented as the mean ± standard error of the mean (SEM). One-way analysis of variance (ANOVA) was used to compare the means, and Fisher’s least significant difference test was applied for multiple comparisons. A value of *p* < 0.05 was considered statistically significant.

## Results

### The comparison of arterial oxygen saturation (SpO_2_)

**Fig. 2:**
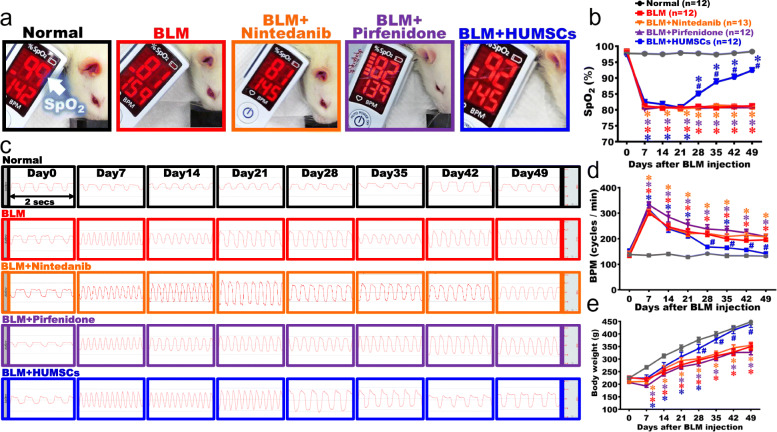
Comparison of lung functions in rats with PF. The photographs reveal that the rats in each group were examined for arterial blood oxygen saturation (SpO_2_) in their forelimbs on day 49 after BLM injury (**a**). Transplantation of HUMSCs increased SpO_2_ in rats with PF when compared to other treatment methods on day 49 (**a**). Weekly records suggested that the SpO_2_ level significantly decreased since day 7 after BLM damage. Except the BLM + HUMSCs group, the trend of reduced SpO_2_ persisted up to day 49 in other treatment groups (**b**). The respiratory frequency was captured within 2 s from day 0 to 49 from each group (**c**). Compared to other treatments, transplantation of HUMSCs alleviated respiratory rates in rats with PF (**d**). Rats were weighed every week for 7 weeks. The results showed that the transplantation of HUMSCs improved body weight of rats with PF (**e**). *n* = 12 or 13 in each group. **✽**: vs the Normal group, *p* < 0.05. **#**: vs the BLM group, *p* < 0.05

The SpO_2_ was assessed using a pulse oximeter to evaluate the lung function of gas exchange. The mean (± SEM) SpO_2_ remained at 98.8 (± 0.4) % on day 49 in the Normal group. The SpO_2_ of the BLM group markedly decreased on day 7 and remained around 81.6 ± 1.4% until day 49, which was significantly lower than that in the Normal group (Fig. 2a, b). For the BLM + Nintedanib, BLM + Pirfenidone, and BLM + HUMSCs groups, the SpO_2_ significantly decreased during day 7 to day 21, which was similar to that in the BLM group. From day 21 to day 49, no increment in SpO_2_ was observed for the BLM + Nintedanib and BLM + Pirfenidone groups. A significant increase in SpO_2_ was observed in the BLM + HUMSCs group since day 28, and the level continuously increased to 92 ± 2.3% on day 49, which was significantly improved as compared with other treatment groups (Fig. 2a, b).

### The comparison of respiratory rate

Breaths per minute (BPM) were counted to estimate lung function. The results of the respiratory function revealed that the respiratory rates remained stable in the Normal group, with 4–5 breaths recorded in 2 s. The respiratory rate marked increased during day 7 to day 49 in the BLM group. For the BLM + Nintedanib, BLM + Pirfenidone, and BLM + HUMSCs groups, the trends of increased respiratory rate were similar to the BLM group during day 7 to day 21. With HUMSCs transplanted on day 21, the BLM + HUMSCs exhibited a reduced respiratory rate since day 28 as compared with other treatment groups. The trend remained until day 49 (Fig. 2c, d).

### The comparison of body weight

The results of body weight showed that the weight in the Normal group gradually increased over time. The BLM group displayed a flatter slope in weight gain since day 7 after BLM injection; although the weight still increased over time, a significant reduction was observed as compared to those in the Normal group, and this trend continued until day 49. For the BLM + Nintedanib, BLM + Pirfenidone, and BLM + HUMSCs groups, the curves of the body weight were similar to those in the BLM group after day 7 as the weight increased in a slower slope and were significantly lower than those in the Normal group. Although the rats in the BLM + Nintedanib and BLM + Pirfenidone groups exhibited an increase in weight from day 7 to day 49, no significant difference was found when comparing to the BLM group, and a significant reduction was seen as compared to the Normal group. The weight of the BLM + HUMSCs group showed a significant increase since day 28 when comparing to those in the BLM group. The weight trend remained until day 39, indicating that transplantation of HUMSCs was superior to other treatments in recovering weight in rats with PF (Fig. 2e).

### The comparison of alveolar space of the left lung

**Fig. 3:**
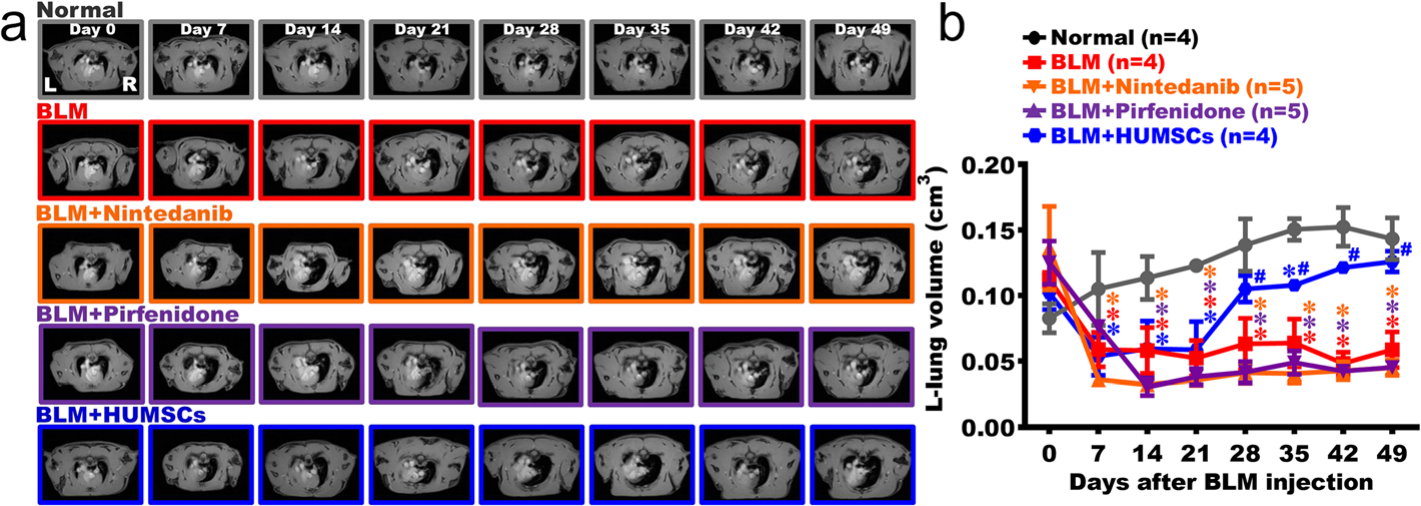
Comparison of the left lung alveolar space in rats with PF. The MRI of rats’ thoracic cavities from the level of the trachea carina is displayed. L indicates the left lung, and R indicates the right lung (**a**). The carina was set as a landmark for image positioning. In addition to the slice containing the carina, two images before and after the carina were collected. These five images were summed for the quantification of the black alveolar space to represent the left-lung alveolar volume of each rat. The results showed that the alveolar volume significantly reduced after BLM damage. Transplantation of HUMSCs effectively increased the alveolar volume. In other treatment groups, the alveolar volume significantly decreased on day 7 after BLM damage, and no improvements were seen until day 49 (**b**). *n* = 4 or 5 in each group. **✽**: vs the Normal group, *p* < 0.05. **#**: vs the BLM group, *p* < 0.05

The changes in left-lung alveolar volume were quantified utilizing an MRI scan**.** With the existence of alveoli in both the left and right lungs, black signals were predominantly seen in the image of the Normal group (Fig. 3a and Supplemental Figure 3). Due to inflammation and white blood cell infiltration, white signals were seen in the left lung of the BLM group on day 7. The alveolar space almost disappeared in the left lung and was mostly occupied by consolidated tissues since day 14; the condition persisted up to day 49 (Fig. 3a, b and Supplemental Figure 4). For the BLM + Nintedanib, BLM + Pirfenidone, and BLM + HUMSCs groups, white signals appeared in the left lung during day 7 to day 21, indicating the loss of alveolar space and replacement with the consolidated tissues. During day 28 and day 49, alveolar space in the left lung was still occupied by the white consolidated tissues (Fig. 3a, b and Supplemental Figure 5–7). However, black alveolar space could be seen since day 28 in the left lung of the BLM + HUMSCs group (Fig. 3a, b).

### The comparison of alveolar structure in the left lung

**Fig. 4:**
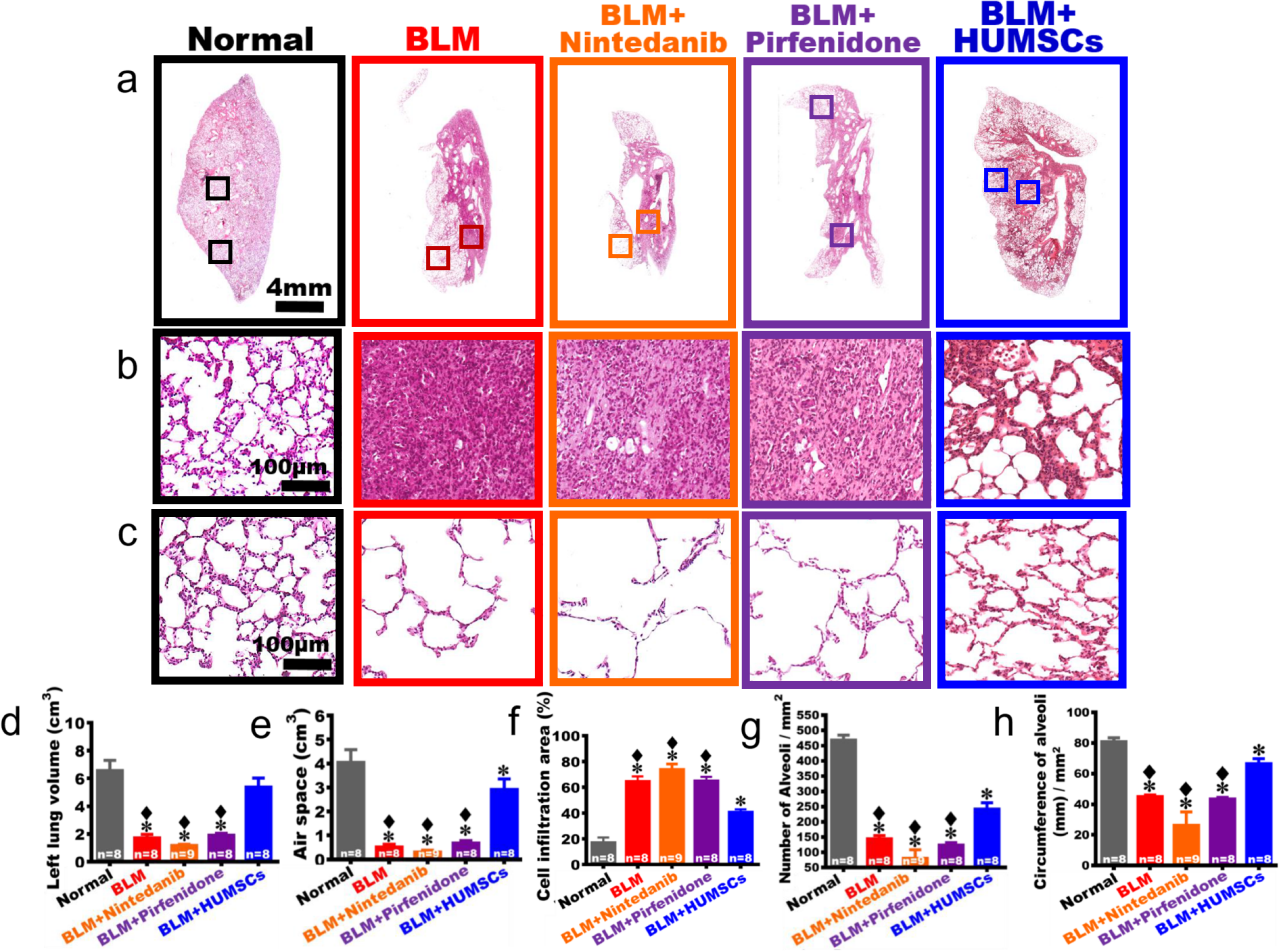
Comparison of the left lung structure in rats with PF. The pictures with a low magnification of left lung sections stained with H&E was obtained from the Normal, BLM, BLM + Nintedanib, BLM + Pirfenidone, and BLM + HUMSCs groups on day 49 (**a**). From high magnification, a massive cell infiltration in the center areas of the left lungs was seen after BLM damage. Transplantation of HUMSCs ameliorated cell infiltration as the alveolar structures were observed (**b**). From high magnification, the alveolar structures were preserved in the outer region of the left lung, albeit the sizes of alveoli varied (**c**). The total volume of left lung was quantified by summing data from all left lung sections, which revealed that transplantation of HUMSCs significantly increased the total left lung volume (**d**), raised the total air space (**e**), and reduced the area infiltrated by inflammatory cells (**f**). The number (**g**) and the circumference (**h**) of alveoli in the unit area in the outer regions of the left lung were further quantified, which showed that transplantation of HUMSCs effectively increased the number and circumference of alveoli in the unit area for gas exchange, while the left lung structures of the rest treatment groups were similar with the BLM group. *n* = 8 or 9 in each group. **✽**: vs the Normal group, *p* < 0.0001. ♦: vs the BLM + HUMSCs group, *p* < 0.0001

On day 49 after BLM injection, the left lung tissues were obtained and subjected to H&E stain. From low to high magnifications, the central areas of the images showed that the alveolar structure was intact in the Normal group, with the connective tissues primarily surrounded the bronchus and rarely appeared among alveoli. In the BLM group, intact alveoli only appeared in the outer regions of the left lung, and the central areas were mostly cell-infiltrated. In the BLM + Nintedanib and BLM + Pirfenidone groups, healthy alveoli were also observed in the outer regions of the left lung, and the central areas were infiltrated by a substantial amount of cells. On the contrary, the area with cell infiltration significantly decreased, and the alveolar space markedly increased in the central area of the BLM + HUMSCs group (Fig. 4a, b). By summing data from all left lung sections stained with H&E, the statistical analyses showed that the left lung volume significantly shrank in the BLM group, in which the volume of alveolar structure decreased, whereas 70% of the left lung was occupied by the consolidated tissues (Fig. 4d–f).

The left lung volume, the air space, and the cell infiltration area in the BLM + Nintedanib and BLM + Pirfenidone groups were similar to the BLM group as no statistical differences were seen (Fig. 4d–f). The left lung volume and the air space in the BLM + HUMSCs group were both comparable with those in the Normal group. In addition, the volume of connective tissues and cell infiltration significantly decreased as compared to those of the BLM group (Fig. 4d–f).

Furthermore, the number and the circumference of alveoli in the unit area in the outer region of the left lung where alveoli remained were quantified to evaluate the effectiveness of gas exchange by alveoli. From low to high magnifications of the H&E stained sections, the alveolar size in the outer region of the left lung was smaller in the Normal group; therefore, the number of alveoli in the unit area was higher, and the total alveolar circumference for the gas exchange was longer. The alveolar size in the outer region in the BLM group from day 7 to day 49 was significantly larger than that in the Normal group, resulting in a decrease in the total number and alveolar circumference of alveoli in the unit area. The number of alveoli and the total alveolar circumference in the unit area of the BLM + Nintedanib and BLM + Pirfenidone groups did not significantly differ from those in the BLM group. For the BLM + HUMSCs group, the number of alveoli and the alveolar circumference in the unit area significantly were increased as compared with those in the BLM group, indicating that transplantation of HUMSCs improves the efficiency of gas exchange (Fig. 4a, c, g, h).

### The comparison of the overall appearance of the left lung

**Fig. 5:**
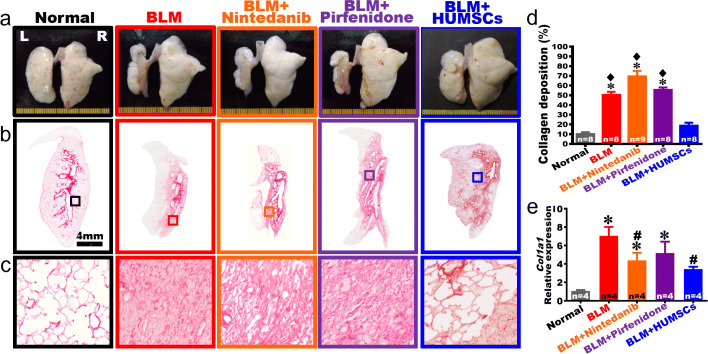
Comparison of gross appearance and fibrosis of left lung in rats with PF. **a** The anterior view of left and right lungs of the Normal, BLM, BLM + Nintedanib, BLM + Pirfenidone, and BLM + HUMSCs groups were obtained on day 49 after BLM damage. Except BLM + HUMSCs group, the overall appearances showed that the left lungs markedly shrank, and alveoli only existed at the outer region of the left lungs on day 49. **b** Subsequently, left lung sections were stained with Sirius red to indicate the location of collagen, which was stained in red. **c** The pictures with higher magnification showed that large red regions appeared in the left lung on day 49 after BLM damage, suggesting disposition of collagen. **d** The percentage of area occupied by collagen in the rats’ left lung was calculated. Transplantation of HUMSCs alleviated the shrinking and fibrosis in rats with PF as compared to those of other treatments. *n* = 8 or 9 in each group. **e** Rat *Col1a1* mRNA was extracted from left lung suspensions and analyzed by quantitative qRT-PCR, demonstrating that a significant decrease in *Col1a1* expression in the BLM + Nintedanib and BLM + HUMSCs groups compared to that in the BLM group on day 49. *n* = 4 in each group. **✽** vs the Normal group, *p* < 0.0001. ♦ vs the BLM + HUMSCs group, *p* < 0.0001. **#** vs the BLM group, *p* < 0.05

On day 49, the overall appearances of the right and left lungs showed that white alveolar structures with intact and smooth alveoli could be seen in the Normal group. The left lung markedly shrunk in the BLM group, with healthy alveoli only presented in the outer region of the left lung and no alveoli but pathological tissues appeared in its central area. Moreover, the pathological tissues in the central area of the left lung displayed dry and flat features. For the BLM + Nintedanib and BLM + Pirfenidone groups, no apparent improvements in the overall appearance of the left and right lungs were observed. However, the volume and the white alveolar regions of the left lung were both markedly preserved in the BLM + HUMSCs group on day 49 (Fig. 5a).

### The comparison of collagen deposition

The left lung sections were stained with Sirius red to label collagen red (Fig. 5b, c). Collage predominantly surrounded the bronchus and blood vessels in the Normal group. The area of collagen stained in red significantly increased in the BLM group. The level of collagen deposition in the BLM + Nintedanib and BLM + Pirfenidone groups did not decrease when comparing to that of the BLM group. The level of collagen deposition in the BLM + HUMSCs was not only lower than those in the BLM, BLM + Nintedanib and BLM + Pirfenidone groups but also comparable with that in the Normal group (Fig. 5b–d). Simultaneously, real-time RT-PCR was used to quantify the level of rat *Col1a1* mRNA in the fresh left lung. The relative expression of rat *Col1a1* mRNA in the BLM, BLM + Nintedanib and BLM + Pirfenidone groups increased significantly compared to that of the Normal group. The relative expression of rat *Col1a1* mRNA in the BLM + Nintedanib and BLM + HUMSCs groups decreased significantly compared to that in the BLM group on day 49 (Fig. 5e).

### The comparison of myofibroblast activation

**Fig. 6:**
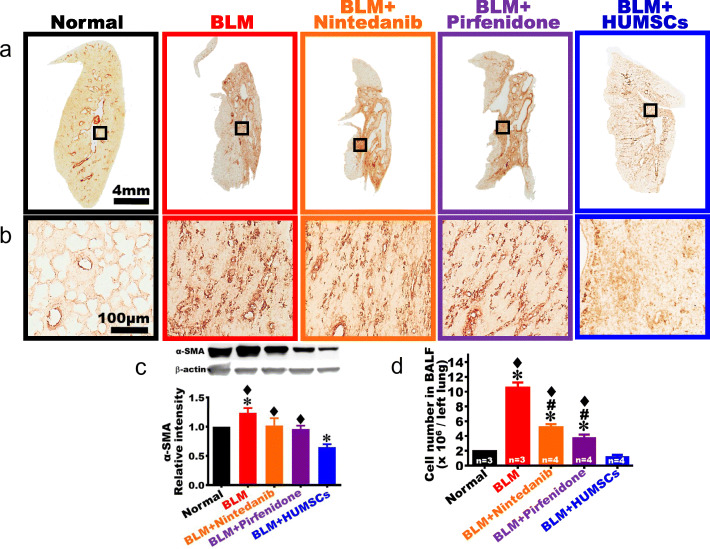
Comparison of the activation of fibroblasts of the left lung in rats with PF. Left lung sections were obtained from the Normal, BLM, BLM + Nintedanib, BLM + Pirfenidone, and BLM + HUMSCs groups on day 49 and were labeled with anti-α-SMA antibody to indicate activated fibroblasts in the left lungs of each study group. From low to high magnifications, a large number of activated fibroblasts appeared in the connective tissues in the BLM, BLM + Nintedanib, and BLM + Pirfenidone groups (**a**, **b**). Furthermore, Western blot was applied to quantify the α-SMA level in the left lung. The results indicated that activated fibroblasts significantly reduced after transplantation of HUMSCs compared to those in other treatment groups (**c**). The cell number in bronchoalveolar lavage was quantified on day 49, which revealed that the cell counts remained higher in the BLM group compared to those in other treatment groups (**d**). *n* = 3 or 4 in each group. **✽**: vs the Normal group, *p* < 0.05. ♦: vs the BLM + HUMSCs group, *p* < 0.05. **#**: vs the BLM group, *p* < 0.0001

Anti-α-SMA antibody was used to identify myofibroblasts (Fig. 6a–c). The images of the Normal group showed that only a very limited number of α-SMA-positive cells existed, primarily surrounded the bronchus and blood vessels. A substantial amount of α-SMA appeared in the connective tissues in the BLM group. The expression level of α-SMA was assessed by Western blotting, which showed that α-SMA significantly increased in the BLM group in contrast to that in the Normal group. The expression level of α-SMA in the BLM + Nintedanib and BLM + Pirfenidone groups showed a decreasing trend, but not a significant amelioration as compared with that in the BLM group (Fig. 6a–c). The expression of α-SMA significantly decreased in the BLM + HUMSCs group as compared with that in the BLM group (Fig. 6a–c).

### The comparison of cell number in bronchoalveolar lavage

Cell number measured in BALF was used to estimate the amplitude of pulmonary inflammation. The results showed that the inflammatory cell counts significantly increased in the BLM group (Fig. 6d). The inflammatory cell counts in the BLM + Nintedanib and BLM + Pirfenidone groups were lower than that in the BLM group but were higher than that in the Normal group. The inflammatory cell count in the BLM + HUMSCs was significantly lower than those in the BLM, BLM + Nintedanib, and BLM + Pirfenidone groups and was comparable with that in the Normal group (Fig. 6d).

### The comparison of macrophage activation and MMP-9 synthesis

**Fig. 7:**
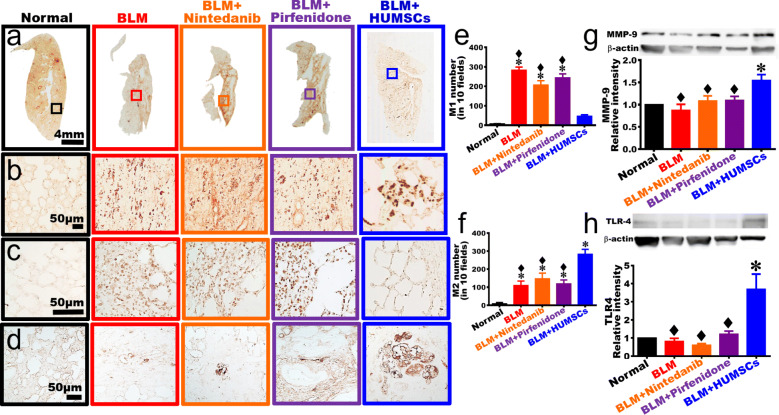
Comparison of the activation of macrophage and the expression of TLR-4 of the left lung in rats with PF. Left lung sections were obtained from the Normal, BLM, BLM + Nintedanib, BLM + Pirfenidone, and BLM + HUMSCs groups on day 49 and were subjected to immunohistochemical stain with anti-ED1 antibody for macrophage labeling. From low to high magnifications, the number of macrophages with a relatively small morphology increased in the BLM group. Transplantation of HUMSCs triggered the activation of macrophage with a relatively bigger size. The patterns of macrophages were similar between the rest treatment groups and the BLM group (**a**, **b**). Next, anti-CD86 antibody was applied to label M1 macrophage, which revealed that the number of M1 macrophages increased in the left lungs of the BLM, BLM + Nintedanib, and BLM + Pirfenidone groups (**c**, **e**). Additionally, anti-CD163 antibody was used to mark M2 macrophage, which showed that the number of M2 macrophages elevated in the left lung of the BLM, BLM + Nintedanib, and BLM + Pirfenidone groups; the number of M2 macrophages significantly increased in the BLM + HUMSCs group (**d**, **f**). Moreover, the expression level of MMP-9 (**g**) and TLR-4 (**h**) in the left lung was quantified with Western blotting, results showed that transplantation of HUMSCs resulted in alternative macrophage activation and increased MMP-9 and TLR-4 expressions in the left lung of rats with PF. *n* = 3 in each group. **✽**: vs the Normal group, *p* < 0.05. ♦: vs the BLM + HUMSCs group, *p* < 0.05

Anti-ED1 antibody was used to identify macrophage (Fig. 7a, b). The results showed that only a limited number of macrophages existed in the Normal group. The number of macrophages increased, with a smaller size in the BLM group. In the BLM + Nintedanib and BLM + Pirfenidone groups, a large number of macrophages with a smaller size substantially appeared in the connective tissues of the left lung. In the BLM + HUMSCs group, macrophages with a larger size were observed in the connective tissues and among the alveolar space of the left lung (Fig. 7a, b). Anti-CD86 antibody was further applied to mark M1 macrophages. The results showed that the number of M1 macrophages was higher in the BLM, BLM + Nintedanib, and BLM + Pirfenidone groups. Transplantation of HUMSCs significantly reduced the number of M1 macrophages (Fig. 7c, e). When using anti-CD163 antibody to label M2 macrophages, it showed that the number of M2 macrophages was scarce in the BLM, BLM + Nintedanib, and BLM + Pirfenidone groups, whereas the number of M2 macrophages was further increased in the BLM + HUMSCs group (Fig. 7d, f).

Western blotting was applied to quantify the expression of matrix metallopeptidase 9 (MMP-9). The results showed that no statistical differences were identified among the Normal, BLM, BLM + Nintedanib, and BLM + Pirfenidone groups. The MMP-9 level markedly elevated in the BLM + HUMSCs group, suggesting transplantation of HUMSCs increased MMP-9 expression, which could help collagen degradation in the fibrotic region of the lung (Fig. 7g).

### The comparison of TLR-4 expression

The protein level of Toll-like receptor-4 (TLR-4) was quantified using Western blotting. The results showed that no statistical significance in TLR-4 expression was observed between the Normal, BLM, BLM + Nintedanib, and BLM + Pirfenidone groups. The TLR-4 content in the BLM + HUMSCs group significantly increased, suggesting that transplantation of HUMSCs elevated TLR-4 expression, which could help the regeneration of type II alveolar epithelial cells in the fibrotic areas of the lung (Fig. 7h).

### The comparison of oxidation and antioxidant expression

**Fig. 8:**
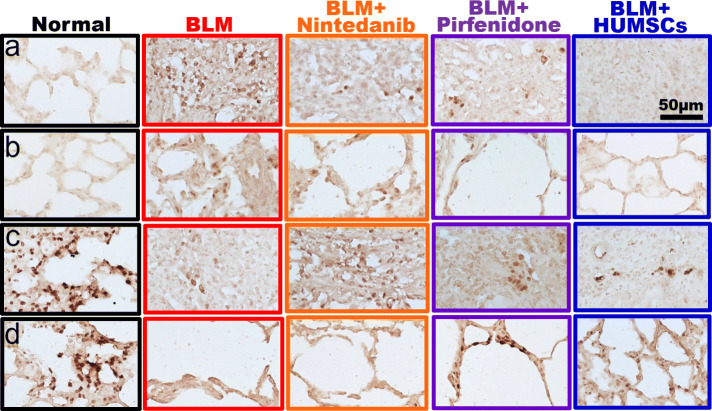
Comparison of the expression of NADPH oxidase and catalase in the left lung in rats with PF. Lung sections were stained with an NADPH oxidase (**a**, **b**) or catalase (**c**, **d**) antibody. Panels **a** and **c** are enlarged images from the central regions, and panels b and d are enlarged images from the peripheral regions. Many of NADPH oxidase-positive cells were found in the scar area in the BLM group. Cell clusters stained robustly with catalase antibody were observed in the Normal group

Lung sections were stained with the NADPH oxidase antibody to examine the distribution and pattern of NADPH oxidase. NADPH oxidase-positive cells were hardly detected in lung sections of the Normal group. NADPH oxidase-positive cells were numerous in the central area with a scar and were scattered in the peripheral area with alveolus in the BLM group. Some NADPH oxidase containing cells were found in the central area, but few with light staining cells existed around the alveoli in the periphery in the groups of BLM + Nintedanib and BLM + Pirfenidone. The pattern of NADPH oxidase staining in the BLM + HUMSCs group was similar to that in the Normal group (Fig. 8a, b).

Lung sections were stained with catalase antibody to examine the distribution of catalase containing cells. Cell clusters with stained intensely were distributed in lung sections of the Normal group. The catalase-positive cells were observed few in number in the central area and were found hardly in the peripheral area in the BLM group. Some catalase-containing cells were existed in the central area but found barely in the periphery in the BLM + Nintedanib group. Catalase-positive cells were sparsely distributed in the central area and in the peripheral area in the BLM + HUMSCs and BLM + Pirfenidone groups (Fig. 8c, d).

## Discussion

In order to compare HUMSCs and anti-fibrotic medications in reversal of PF, initiation of consecutive pirfenidone/nintedanib administration or single intratracheal transplantation of 2.5× 10^7^ HUMSCs was performed on day 21 post-BLM injection. Our results showed that transplanted HUMSCs can effectively reverse PF.

Nintedanib could effectively suppress inflammation, angiogenesis, and the activities of fibroblasts [16, 17]. The results of clinical trials showed that nintedanib reduced the rate of decline in pulmonary function in patients with mild to moderate lung function impairment [19–24]. As for pirfenidone, the daily administration of 300 mg/kg pirfenidone was initiated started from 2 weeks after BLM injury for 4 weeks. The results demonstrated that pirfenidone increased the mRNA expressions of anti-oxidative factors, as well as decreased inflammation and fibrosis in the lung [25]. According to the clinical study results of CAPACITY trials, ASCEND trial, and other phase 3 trials, pirfenidone could diminish the reduction of forced vital capacity, reduced the frequency of acute exacerbation, and increased exercise endurance in patients with PF [26–28]. Currently, a combination of nintedanib and pirfenidone was superior to the nintedanib alone in reducing the loss of lung function; however, as the magnitude of side-effects is greater when combining the two medications, it is less frequently implemented in clinical practice [29].

Nintedanib and pirfenidone reduced fibrotic gene expression including *Col1a1* and *Fibronectin* in murine and human 3D-lung tissue cultures as well as primary murine alveolar epithelial type II cells [30]. Nintedanib and pirfenidone are approved medications known to decrease PF progression. In the present study, nintedanib or pirfenidone administration was initiated at the end of week 3 after BLM damage; at that time, collagen deposition exhibited a considerable enhancement. Our results indicated that nintedanib or pirfenidone could not restore or reverse the damaged lung tissues when pulmonary damage reached a stable and irreversible PF status. Nevertheless, bronchoalveolar lavage cell counting revealed that the cell number in the nintedanib or pirfenidone groups was significantly lower than the BLM group, indicating nintedanib or pirfenidone reduced inflammatory responses in the chronic stage. Moreover, the relative expression of rat *Col1a1* mRNA in the BLM + Nintedanib and BLM + HUMSCs groups decreased significantly compared to that in the BLM group on day 49. However, the distribution and pattern of catalase-positive cells are different between the groups of BLM + Nintedanib and BLM + Pirfenidone, resulting from the diverse mechanisms to treat lung fibrosis of these two medications.

In most studies, stem cells were transplanted prior to fibrogenesis in the lung, as the researchers primarily focused on alleviating the inflammation of the lung and preventing the development of PF [31–34]. We previously discovered that xenografted HUMSCs did not differentiate into functional lung cells but secreted a variety of cytokines (human FGF-6 and IGF-1) and HA that effectively reversed PF [9]. Three underlying therapeutic mechanisms exist. First, HUMSCs inhibited inflammation, reduced myofibroblast activity, and prevented more collagen synthesis. Second, HUMSCs activated the host’s macrophages to synthesize MMP-9, which degraded the existing collagen. Third, HUMSCs promoted the expression of TLR-4 in the host’s alveolar epithelial cells and triggered the signal of HA-TLR-4 for lung regeneration [9]. Similar opinions were supported by Glasser et al. [35]. In the present study, 2.5× 10^7^ HUMSCs were transplanted into the lung of a rat with PF on day 21 after BLM damage. HUMSCs transplantation reduced inflammatory response, as demonstrated by bronchoalveolar lavage cell counting on day 49. Immunostaining using the anti-α-SMA antibody showed that HUMSCs transplantation effectively reduced fibroblast activation. As a result, new ECM synthesis and accumulation were significantly diminished and halted.

In this study, Sirius red staining revealed that HUMSCs transplantation reduced collagen deposition. Immunostaining using the anti-ED1 antibody showed that HUMSCs transplantation triggered a large number of macrophage activation, especially M2 macrophages. Additionally, the transplantation of HUMSCs may also stimulate MMP-9 concentration. We previously found that the transplantation of HUMSCs promotes M2 macrophage to synthesize and secrete MMP-9 to facilitate the degradation of extracellular matrix, such as collagen [9]. Cabrera et al. also had the same conclusion regarding MMP-9 in macrophage [36].

In the present study, HUMSCs transplantation into rats with PF increased the TLR-4 content in the lung from the results of Western blotting. Activation of TLR-4 plays a vital role in the regeneration and reconstitution of alveolar epithelial cells [37, 38]. Furthermore, the binding of hyaluronan and TLR-4 could also promote macrophage to differentiate into M2 phenotypes [39]. We previously discovered that the transplantation of HUMSCs robustly increased the TLR-4 protein around the alveolar circumference and in the M2 macrophage [9].

Increasing evidences showed that HUMSCs exhibited long-term survival within different organs in rats and, therefore, could serve as an excellent cell source suitable for xenograft [40–48].

Because the number of transplanted stem cells is considered as an important issue to regeneration, HUMSCs were implanted into different target organs depending on the diseases [40–48]. In this study, HUMSCs were implanted intratracheally to allow most transplanted stem cells migrating into the damaged lung. Clinically, it is always not recommended to use invasive deliveries. Therefore, we should test how many HUMSCs need to be transplanted by the intravenous route (non-invasive), how often, and how many times need to transplant to reverse PF before clinical trials.

## Conclusions

HUMSCs transplantation could effectively reverse lung fibrosis and may provide a new option for treatment for the patients with existing *PF* and COVID-19 sequela.

## Supplementary Information

**Additional file 1: Supplemental Figure 1.** Surface markers and differentiation ability of HUMSCs in vitro. (A) Flow cytometry analyses of surface markers of HUMSCs in vitro. HUMSCs were cultured for 10 passages and then labeled with CD44, CD105 and HLA-DR antibodies. White areas represent negative controls and red areas represent the specific binding for indicated antigens. The results revealed that HUMSCs transplanted into rats were positive for CD44 and CD105 but negative for HLA-DR. (B) Multipotency of HUMSCs. HUMSCs could differentiate into alkaline phosphatase-positive osteogenic cells (left panel) and NeuN-positive neuronal cells (right panel) when cultured in differentiation medium. Scale bar: 100 μm. **Supplemental Figure 2.** Sectioning methodology of paraffin-embedded lung tissues. A consecutive of 10 slices numbered 1 to 10 were placed on different slides for different immunostainings. Taking the Normal group as an example, lung slices numbered 1 to 10 were placed on slides lettered A, B, C, D, E, F, G, H, I, and J. Slices numbered 11 to 30 were discarded (a total of 20 slices). Similarly, slices numbered 31 to 40 were placed on slides lettered from A to J. The next 20 slices were also discarded. The same procedures were repeated until the entire lung tissue was completely sectioned. Therefore, the slices in column A (numbered 1, 31, 61, 91, and 121-871) were all subjected to H&E statin to assess the histopathology of the tissue; lung slices in column B (numbered 2, 32, 62, 92, 122-872) were stained with Sirius red for evaluation of tissue fibrosis; lung slices in column C (numbered 3, 33, 63, 93, 123-873) were subjected to immunohistochemical staining with anti-α-SMA antibody to examine the changes of myofibroblasts; lung slices in column D (numbered 4, 34, 64, 94, 124-874) were immunostained with anti-ED1 antibody to label macrophages. Rows A①-J①, A②-J②, and A③-J③ represent the outermost region of each lobe in the left lung; rows A⑥-J⑥ and A⑦-J⑦ represent the region close to hilum in the left lung. The number of lung slices obtained varied in different groups (range: 330-880 slices). The rest columns (F-J) were preserved as spares. **Supplemental Figure 3.** MRI scans of rats’ thoracic cavities in the Normal group. In the Normal group, MRI scans of rats’ thoracic cavities were performed horizontally from rostral to caudal in an interval of 1.5 mm. On Day 0, for example, the carina of trachea was used as a landmark for image positioning (A3). In addition to the slice containing the carina, two slices before (A1-A2) and after (A4-A5) the carina were collected. These five images were summed for quantification of the black alveolar space to represent the left-lung alveolar volume of each rat. L indicates the left side of the body and R indicates the right. A1-A5 represent Day 0, B1-B5 represent Day 7, C1-C5 represent Day 14, D1-D5 represent Day 21, E1-E5 represent Day 28, F1-F5 represent Day 35, G1-G5 represent Day 42, and H1-H5 represent Day 49. **Supplemental Figure 4.** MRI scans of the rats’ thoracic cavities in the BLM group. In the BLM group, MRI scans of rats’ thoracic cavities were performed horizontally from rostral to caudal in an interval of 1.5 mm. The carina of trachea was used as a landmark for image positioning (A3, B3, C3, D3, E3, F3, G3, and H3). In addition to the slice containing the carina, two slices before and after the carina were collected. These five images were summed for quantification of alveolar volume in each rat. **Supplemental Figure 5.** MRI scans of the rats’ thoracic cavities in the BLM+Nintedanib group. Five MRI scans of the thoracic cavities in the BLM+Nintedanib group are presented. **Supplemental Figure 6.** MRI scans of the rats’ thoracic cavities in the BLM+ Pirfenidone group. Five MRI scans of the thoracic cavities in the BLM+ Pirfenidone group are presented. **Supplemental Figure 7.** MRI scans of the rats’ thoracic cavities in the BLM+ HUMSCs group. Five MRI scans of the thoracic cavities in the BLM+ HUMSCs group are presented.

## Data Availability

All the data supporting the findings will be made public and can be shared by contacting the corresponding authors THChen and YSFu.

## Abbreviations

PF: Pulmonary fibrosis
BLM: Bleomycin
HUMSCs: Human umbilical mesenchymal stem cells
PDGF: Platelet-derived growth factor
VEGF: Vascular endothelial growth factor
FGF: Fibroblast growth factor
SD: Sprague-Dawley
HE staining: Hematoxylin and eosin staining
α-SMA: α-Smooth muscle actin
TLR-4: Toll-like receptor-4
MMP-9: Matrix metallopeptidase 9
